# Genomic and Functional Characterization of *Pseudomonas shiyinii* sp. nov. ST4 Reveals Conserved Biocontrol Mechanisms Against Sugarcane Smut

**DOI:** 10.3389/fmicb.2026.1783009

**Published:** 2026-05-20

**Authors:** Yonglin Li, Shiyin Liu, Changlong Yang, Changqing Chang, Ming Hu, Lianhui Zhang, Jianuan Zhou, Xiaofan Zhou

**Affiliations:** National Key Laboratory of Green Pesticide, Guangdong Provincial Key Laboratory of Microbial Signals and Disease Control, Engineering Research Center of Biological Control, Ministry of Education, Integrative Microbiology Research Center, South China Agricultural University, Guangzhou, China

**Keywords:** biological control, glucose dehydrogenase, indole-3-acetic acid, novel species, *Pseudomonas*, sugarcane smut

## Abstract

**Introduction:**

*Pseudomonas* sp. ST4 is a potent biocontrol agent against sugarcane smut disease, acting primarily by inhibiting the sexual mating of the fungal pathogen *Sporisorium scitamineum*. However, the genetic basis of its antifungal activity has remained unexplored.

**Methods:**

To elucidate the genetic basis of ST4’s biocontrol activity, we obtained its complete genome sequence and conducted comprehensive genomic, phylogenetic, and chemotaxonomic analyses. We additionally performed a genome-wide Tn5 transposon mutagenesis screen to identify genes critical for its inhibitory activity, followed by a broader phylogenomic analysis to assess the evolutionary conservation of these traits across the *Pseudomonadaceae* family.

**Results:**

Based on our analyses, we delineated ST4 as a novel species, *Pseudomonas shiyinii* sp. nov. The 5.14-Mb genome harbors numerous genes and biosynthetic gene clusters associated with plant-beneficial traits. The Tn5 mutagenesis screen revealed that the pyrroloquinoline quinone (PQQ)-dependent glucose dehydrogenase (Gcd) pathway is essential for the inhibitory activity of ST4, with 78% of loss-of-function mutants mapping to the *pqq* or *gcd* genes. We further demonstrated that acidification of the environment mediated by the PQQ-Gcd system in ST4 inhibits *S. scitamineum* mating—a critical step in pathogenicity—without compromising haploid sporidial viability. In addition to this primary mechanism, the ST4 genome contains complete biosynthesis pathways for the phytohormone indole-3-acetic acid (IAA), which may also serve as a precursor for the previously identified active metabolite indole-3-carbaldehyde (ICHO), a specific inhibitor of fungal sexual mating. *Phylogenomic* analysis showed that while the PQQ-Gcd system is broadly conserved across *Pseudomonadaceae*, the capacity for IAA/I3C production is more specialized, confined to approximately 21% of Pseudomonas species.

**Discussion:**

Together, our findings decipher the molecular mechanism of a widely conserved biocontrol trait and establish *P. shiyinii* ST4 as a promising, ecologically adaptable candidate for sustainable crop protection.

## Background

The genus *Pseudomonas* represents a vast reservoir of microbial talent, comprising species renowned for their metabolic versatility and ecological ubiquity (Palleroni, 2015). These bacteria are frequently isolated from diverse environments, including the rhizosphere, where they play pivotal roles as agents of biocontrol, plant growth promotion, and bioremediation (Weller, 2007; Mercado-Blanco and Bakker, 2007). Notable examples include *P. protegens* and *P. fluorescens*, which act as effective biocontrol agents by suppressing soil-borne pathogens through antimicrobial compounds like 2,4-diacetylphloroglucinol (DAPG) and hydrogen cyanide (Keel et al., 1992; Ramette et al., 2011). Species such as *P. chlororaphis* and *P. putida* promote plant growth by secreting phytohormones like indole-3-acetic acid (IAA) and siderophores that sequester iron, making it unavailable to pathogens (Patten and Glick, 2002; Dimkpa et al., 2009). Furthermore, members like *P. aeruginosa* and *P. stutzeri* are employed in bioremediation for their remarkable ability to degrade a wide range of environmental pollutants, including hydrocarbons and heavy metals (Wasi et al., 2013). This multifunctional efficacy stems from a sophisticated arsenal of mechanisms, including the production of a wide array of secondary metabolites, lytic enzymes, and siderophores that collectively suppress phytopathogens and bolster plant health (Haas and Fago, 2005).

A formidable challenge to global sugarcane production is sugarcane smut, a devastating fungal disease caused by the basidiomycete *Sporisorium scitamineum* (Rajput et al., 2021). The pathogenicity of *S. scitamineum* is contingent upon the sexual mating of compatible haploid sporidia to form infectious dikaryotic hyphae, which penetrate the host plant (Schirawski et al., 2010; Yan et al., 2016). This disease leads to significant yield losses, often exceeding 30% ~ 50% in susceptible varieties, and poses a persistent threat to the sustainability of the sugar industry (Croft and Magarey, 2008). Current management strategies primarily rely on resistant cultivars, a solution constrained by lengthy breeding cycles and the emergence of new pathogen races (Shen et al., 2014). Chemical control is largely ineffective due to the endophytic growth habit of the fungus and the protective sugarcane cuticle, highlighting a critical need for sustainable and effective alternatives such as biocontrol (Bhuiyan et al., 2012).

A promising candidate for the biocontrol of sugarcane smut is the bacterial strain *Pseudomonas* sp. ST4, isolated from the rhizosphere and originally identified as *P. guariconensis* (Liu et al., 2017). ST4 exhibits a distinctive mode of action by specifically disrupting the sexual reproduction of *S. scitamineum*. It secretes glucose-dependent metabolites that potently inhibiting mating between compatible sporidia, reducing dikaryotic hyphal formation without killing the haploid cells (Liu et al., 2018). Bioassay-guided fractionation identified key bioactive compounds, including indole-3-carbaldehyde, which blocks mating at low concentrations. The practical relevance of this antagonism was confirmed by a considerable increase in smut control efficacy when ST4 was co-applied with glucose both *in vitro* and under greenhouse conditions (Liu et al., 2017). Despite these promising phenotypic observations, the genetic and molecular basis underlying this targeted antifungal activity remained uncharacterized.

The efficacy of biocontrol agents is profoundly influenced by carbon source availability, which modulates the production of antagonistic compounds through both nutritional and regulatory mechanisms (Duffy and Fago, 1999). Simple sugars such as glucose can act as preferred carbon sources that fuel the energy-intensive synthesis of secondary metabolites. Furthermore, sugar metabolism can directly regulate biocontrol traits; for example, bacterial oxidation of glucose to gluconic acid via the glucose dehydrogenase (GDH)/pyrroloquinoline quinone (PQQ) system acidifies the microenvironment, creating conditions unfavorable to many fungal pathogens (Cheng et al., 2015; Zamanzadeh-Nasrabadi et al., 2023; Xu et al., 2025). This mechanism, alongside carbon catabolite repression (CCR) where preferred sugars suppress metabolic pathways for alternative substrates, allows bacteria to dynamically tailor their antifungal arsenal in response to nutrient availability (Görke and Stülke, 2008).

The biosynthesis of antimicrobial indole derivatives is a well-characterized trait in many plant-beneficial bacteria, particularly within *Pseudomonas* (Liu et al., 2018). These compounds primarily originate from tryptophan metabolism via conserved pathways such as the indole-3-acetamide (IAM) and indole-3-pyruvate (IPyA) routes, which are best known for producing the phytohormone IAA (Spaepen and Vanderleyden, 2011). Enzymatic modifications, such as those catalyzed by aldehyde dehydrogenases and monooxygenases, further diversify this repertoire into various antifungal aldehydes and acids (Duca et al., 2014). For instance, certain pseudomonads convert tryptophan to indole-3-carbaldehyde (I3C), a metabolite known to interfere with fungal development and quorum sensing in other systems, showcasing a strategic redeployment of phytohormone precursors for pathogen suppression (Liu et al., 2018; Lee and Lee, 2010).

Building upon the established biocontrol efficacy of ST4, this study aimed to elucidate the genetic foundations of its antifungal mechanism. We performed high-coverage whole-genome sequencing to precisely define the taxonomic identity of ST4 and provide a complete genetic blueprint for functional analysis. Subsequent genome mining was conducted to identify secondary metabolite biosynthetic gene clusters (SMBGCs) potentially responsible for its known and novel antimicrobial compounds. Furthermore, a genome-wide Tn5 transposon mutagenesis screen was implemented to directly link genetic determinants to the loss of antifungal activity against *S. scitamineum*. This integrated genomic and genetic approach bridges the gap between genotype and phenotype, unveiling the key mechanisms that empower ST4 to disrupt fungal pathogenesis and solidifying its potential as a novel biocontrol agent.

## Results

### Genome sequencing and analysis of ST4

**Figure 1:**
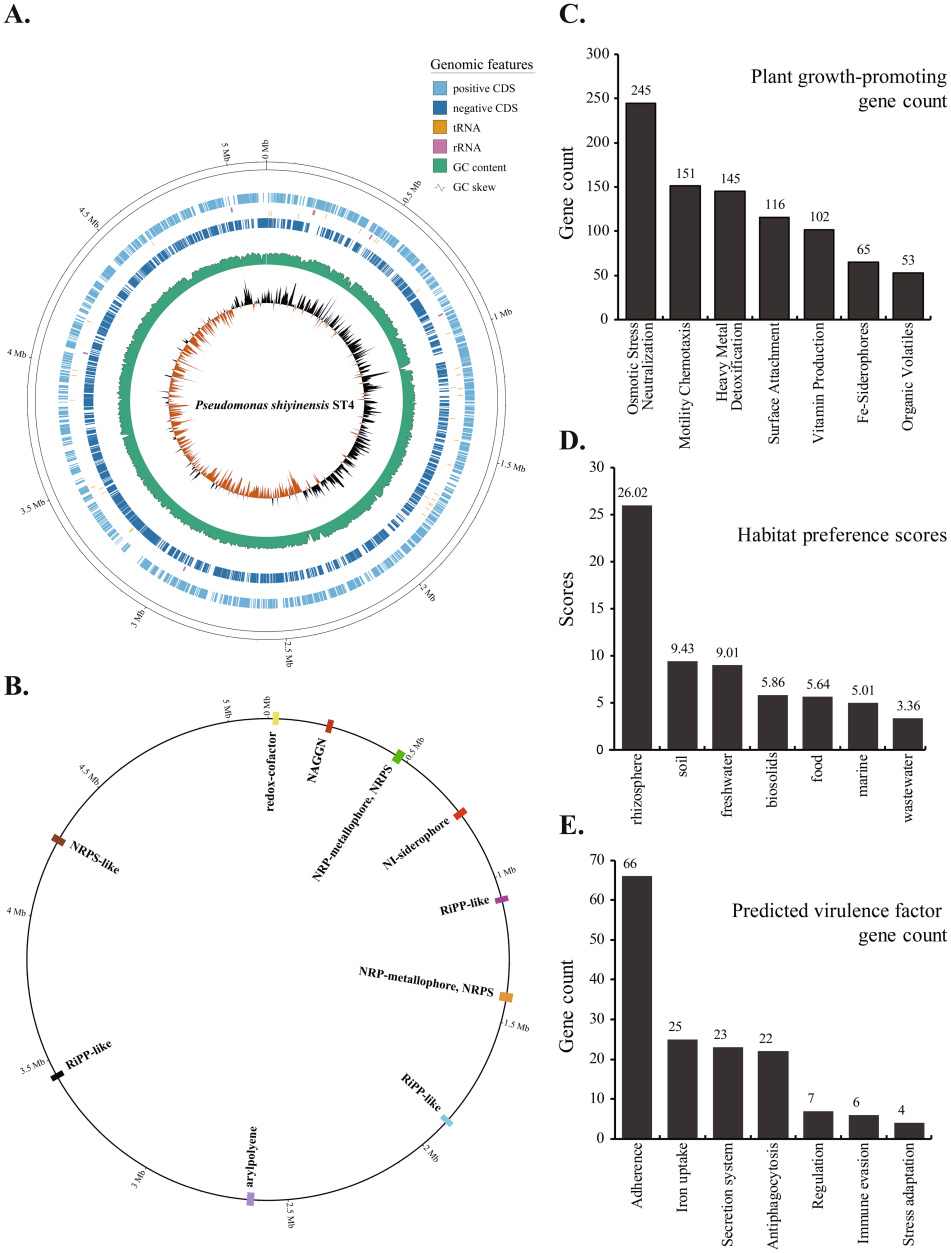
Genomic features and functional potential of *Pseudomonas shiyinensis* ST4. **(A)** Circular map of the complete ST4 chromosome. From outer to inner rings: 1, Genomic coordinates (Mb); 2, protein-coding genes on the forward strand; 3, rRNAs; 4, tRNAs; 5, protein-coding genes on the reverse strand; 6, GC content; and 7, GC skew. **(B)** Genomic locations of secondary metabolite biosynthetic gene clusters (BGCs) predicted by antiSMASH 8. **(C)** The abundance of plant growth-promoting (PGP) genes identified by PGPg-finder, categorized by their primary functional role. **(D)** Habitat preference prediction for ST4 based on ProkAtlas analysis. The bar plot shows the preference scores for different environmental niches, with the rhizosphere and soil being the most likely habitats. **(E)** Putative virulence factors identified in the ST4 genome using VFanalyzer and the Virulence Factor Database (VFDB).

Despite the well-documented biocontrol efficacy of *Pseudomonas* sp. ST4 against sugarcane smut, the absence of a complete genome sequence has limited a deeper understanding of its molecular mechanisms and hindered efforts to fully exploit its biocontrol potential. To bridge this gap, we performed whole-genome sequencing of ST4 using a hybrid approach combining Illumina short-read and PacBio long-read technologies. This generated 473.70 Mb (1.89 million read pairs) and 823.29 Mb (128,682 reads; read length N50: 8,722 bp) of sequencing data, respectively. Hybrid assembly of these data yielded a complete, circular chromosome of 5,135,208 bp with a GC content of 63.57 mol% (Figure 1A; Table 1). The assembly exhibited exceptional completeness, with 99.94% of Illumina and 98.47% of PacBio reads mapping successfully to the genome. BUSCO and CheckM assessments further supported near perfect levels of genome completeness (>99%) and minimal contamination (0.11%). Genome annotation predicted 4,484 protein-coding genes, 19 rRNAs, 73 tRNAs, and four ncRNAs (Table 1).

**Table 1:** Genome characteristics of ST4.

Strain	ST4^T^
Assembly size (bp)	5,135,208
G + C content (%)	63.57
Number of scaffolds	1
Scaffold N50 (bp)	5,135,208
Number of protein-coding genes	4,484
Completeness (%)	99.9
Number of pseudogenes	49
Number of rRNAs	19
Number of tRNAs	73
Number of ncRNAs	4

To investigate the molecular basis of ST4’s biocontrol capacity, we systematically analyzed its genome for specialized metabolic pathways and plant-beneficial traits. AntiSMASH analysis identified ten secondary metabolite biosynthetic gene clusters (SMBGCs), including three non-ribosomal peptide synthetase (NRPS) clusters, three ribosomally synthesized and post-translationally modified peptide (RiPP) clusters, and single clusters for aryl polyene, N-acetylglutaminylglutamine amide (NAGGN), NRPS-independent siderophore (NI-siderophore), and redox-cofactor production (Figure 1B; Supplementary Table S1). The NI-siderophore cluster showed 100% similarity to vibrioferrin biosynthesis clusters, while five other clusters exhibited limited similarity to BGCs responsible for producing compounds such as APE Vf, lankacidin C, nematophin, and Pf-5 pyoverdine. Complementary analysis using PGPg-finder and the PLaBAse database identified 1,111 plant growth-promoting genes, prominently enriched in functional categories including “Osmotic Stress Neutralization” (245 genes), “Motility Chemotaxis” (151 genes), “Heavy Metal Detoxification” (145 genes), “Surface Attachment” (116 genes), and “Vitamin Production” (102 genes) (Figure 1C; Supplementary Table S2). Habitat prediction via ProkAtlas, which correlates metagenome-derived 16S rRNA sequences with prokaryotic habitats, indicated that ST4 and its close relatives might adapt to diverse environments, with the rhizosphere (preference score: 26.02), soil (preference score: 9.43), and freshwater (preference score: 9.01) being the most favorable (Figure 1D; Supplementary Table S3).

To evaluate the biosafety and potential pathogenic risks of ST4, we systematically screened its genome for virulence factors using the VFDB database and VFanalyzer. This analysis identified 165 putative virulence-associated genes, including 66 proteins related to adherence, 25 proteins related to iron uptake, 23 proteins related to secretion system, 22 proteins related to alginate biosynthesis and regulation, and 29 proteins involved in various other processes such as stress adaptation, biofilm formation, and efflux pump (Figure 1E; Supplementary Table S4). Notably, ST4 encodes a complete Type VI Secretion System (T6SS) but lacks the Type III Secretion System (T3SS) and its associated effectors (T3Es), which are primary virulence determinants in many bacterial phytopathogens.

### Genomic and chemotaxonomic analyses define ST4 as a novel *Pseudomonas* species

Although *Pseudomonas* sp. ST4 was initially classified as *P. guariconensis* based on 16S rRNA sequence analysis, its GC-content is 2% higher than that of *P. guariconensis* PCAVU11^T^, a notable deviation for strains of the same species. A comprehensive genome-based taxonomic reassessment using GTDB-Tk also challenged this designation, placing ST4 within the “*Pseudomonas_E putida_K*” cluster, which comprises four genomes (GCF_002094795.1, GCF_020151015.1, GCF_035615335.1, GCF_040984105.1) in the GTDB RS226 release. Although the records of these genomes in the NCBI genome database suggest that they all have *P. guariconensis* LMG 27394 (GCF_900102675.1) as their closest type strain match, the Average Nucleotide Identity (ANI) values are only around 88%, well below the accepted 95% threshold for species delineation. Our systematic comparison with all representative and type strain genomes of *Pseudomonas* confirmed that *P. guariconensis* remains the closest match to ST4, albeit with a low ANI value of 86.93% (Table 2). This finding was further corroborated by digital DNA–DNA hybridization (dDDH) analysis, which yielded values ≤33.2% between ST4 and *P. guariconensis* or other *Pseudomonas* species—substantially beneath the 70% species boundary (Table 2). At the same time, we identified in the NCBI genome database six *Pseudomonas* genomes that exhibit high genomic relatedness to ST4, with ANI values between 96.02% ~ 97.76% and dDDH values between 68.4% ~ 81.9%, indicating that they belong to the same species (Table 2).

**Table 2:** Average Nucleotide Identity (ANI) and digital DNA–DNA hybridization (dDDH) values between ST4 and genomes of selected *Pseudomonas* strains.

Strain	ANI (%)	dDDH (%)
“*Pseudomonas putida*” 19NY05SH04	97.76	81.9
“*Pseudomonas putida*” PA1	97.66	80.9
“*Pseudomonas juntendi*” H4	97.54	80.4
“*Pseudomonas putida*” DZ-C18	97.52	80
“*Pseudomonas putida*” 19MO01SH06-1	96.18	69.4
*Pseudomonas* sp. p1(2021b)	96.02	68.4
*Pseudomonas guariconensis* NY14324	86.93	33.2
*Pseudomonas peradeniyensis* BW13M1	85.90	31.3
*Pseudomonas mosselii* PtA1	85.80	30.8
*Pseudomonas muyukensis* COW39	85.77	30.6
*Pseudomonas faucium* BML-PP048	85.62	30.6
*Pseudomonas entomophila* L48	85.53	30.5
*Pseudomonas xantholysinigenes* RW9S1A	85.50	30.2
*Pseudomonas sichuanensis* B21-027	85.48	30.3
*Pseudomonas maumuensis* COW77	85.44	30.3
*Pseudomonas parasichuanensis* BML-PP020	85.29	30.2
*Pseudomonas pudica* 20-MO00628-0	85.14	29.9

**Figure 2:**
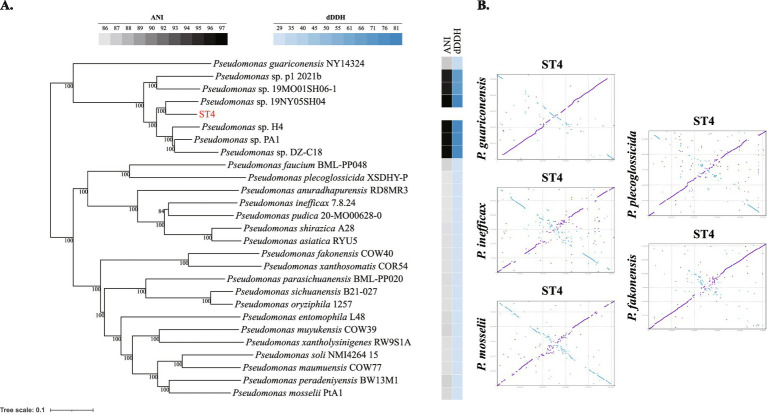
Phylogenomic analysis and genomic comparison of *Pseudomonas shiyinensis ST4* and selected *Pseudomonas* strains. **(A)** Genome-based phylogenetic tree inferred by tANI_tools, showing the evolutionary relationship between strain ST4, its six closest genomic relatives (with ANI values above 95%), and representative genomes of the most closely related *Pseudomonas spe*cies (based on ANI values). Bootstrap values (based on 1,000 replicates) are shown at branch nodes. The average nucleotide identity (ANI) and digital DNA–DNA hybridization (dDDH) values between ST4 and the other strains are indicated, which are significantly below the species delineation thresholds of 95 and 70%, respectively. **(B)** Genome-to-genome dot plots comparing ST4 against five related *Pseudomonas* species.

Phylogenomic reconstruction firmly supports these results: ST4 and these six strains form a distinct, monophyletic clade, with *P. guariconensis* as its closest relative. Both groups are clearly divergent from other established *Pseudomonas* species (Figure 2A). Notably, even between ST4 and its closest relative, *P. guariconensis*, considerable structural variations were observed, further confirming their status as distinct species (Figure 2B; Table 3). The extent of these genomic rearrangements increased with phylogenetic distance, as comparisons with species like *P. plecoglossicida, P. inefficax, P. fakonensis*, or *P. mosselii* showed a more pronounced disruption of colinearity, characterized by a higher frequency of genomic inversions and a greater total length of sequence involved in structural variations (Figure 2B; Table 3).

**Table 3:** Genome structural variations between the ST4 and selected *Pseudomonas* species.

SV information	*P. guariconensis*	*P. plecoglossicida*	*P. inefficax*	*P. fakonensis*	*P. mosselii*
Deletions	133	134	86	132	105
Insertions	110	114	108	198	99
Duplications	2	1	3	3	3
Contractions	0	0	0	0	0
Inversions	13	24	23	18	33
Translocations	0	0	0	0	0
Total SVs	258	273	220	351	240
Total length (bp)	1,981,662	3,107,256	3,104,742	3,069,283	4,138,268

To further establish the taxonomic position of strain ST4, we conducted a comprehensive chemotaxonomic analysis. The respiratory quinone was identified as ubiquinone Q-9, consistent with members of the genus *Pseudomonas*. Polyamine analysis revealed the presence of spermidine, putrescine, and cadaverine (Supplementary Figure S1A). The polar lipid profile comprised diphosphatidylglycerol (DPG), phosphatidylethanolamine (PE), phosphatidylglycerol (PG), and an unidentified lipid (Supplementary Figure S1B). Notably, the cellular fatty acid profile of ST4 exhibited significant differences from its closest relative, *P. guariconensis*. The major components (≥10%, same below) in ST4 were C_16:0_ (30.50%), C_17:0_ cyclo (23.48%) and C_19:0_ cyclo ω8c (12.28%) (Supplementary Table S5), a composition distinct from that reported for *P. guariconensis* (C_16:0_, 25.7%; C_18:1_ ω7c, 20.4%; C_17:0_ cyclo, 11.5%; and C_16:1_ ω7c/C_15:0_ iso 2-OH in summed feature 3, 10.8%).

Collectively, the genomic, phylogenetic, and chemotaxonomic data conclusively demonstrate that ST4 represents a novel species within the genus *Pseudomonas*. We propose the name *Pseudomonas shiyinensis* sp. nov. in honor of the late Dr. Shiyin Liu, who first isolated and characterized this promising biocontrol strain.

### Tn5 mutant screening identifies PQQ-Gcd-mediated acidification as a key biocontrol mechanism

**Figure 3:**
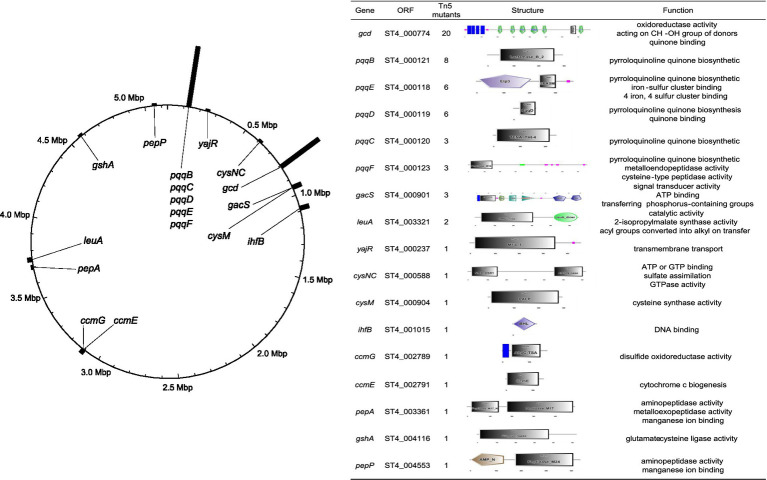
Genome-wide Tn5 mutagenesis screen identifies the PQQ-Gcd pathway as essential for the inhibitory activity of ST4. The figure provides a circular summary of the Tn5 insertion sites mapped from mutants that lost the ability to inhibit *S. scitamineum mat*ing. The ring represents the circular chromosome of *P. shiyinensis ST4*. The bars protruding outward indicate the locations and relative frequencies of Tn5 insertions across the genome. Gene names and functional annotations for the disrupted loci are displayed in the center.

To elucidate the molecular mechanism underlying ST4’s inhibition of *S. scitamineum* sexual mating, we generated a random Tn5 transposon insertion mutant library. From approximately 40,000 mutants screened, we identified 109 strains exhibiting completely abolished or significantly reduced ability to inhibit the mating between *S. scitamineum* MAT-1 and MAT-2 sporidia. The Tn5 insertion sites for 59 mutants were successfully mapped using high-efficiency thermal asymmetric interlaced PCR (hiTAIL-PCR), revealing disruptions in 17 distinct protein-coding genes (Figure 3). Strikingly, the insertions were highly concentrated in two specific genomic regions: 26 mutants had insertions within the *pqqBCDEAF* operon (ST4_000118 to ST4_000123), which is essential for the biosynthesis of the cofactor pyrroloquinoline quinone (PQQ), and 20 mutants had an insertion in *ST4_000774*, encoding the PQQ-dependent glucose dehydrogenase (Gcd) (Figure 3). In total, 78% (46/59) of the mutants with mapped insertions exhibited disruptions in the PQQ-Gcd pathway, strongly suggesting that glucose dehydrogenase activity is central to the biocontrol function of ST4.

**Figure 4:**
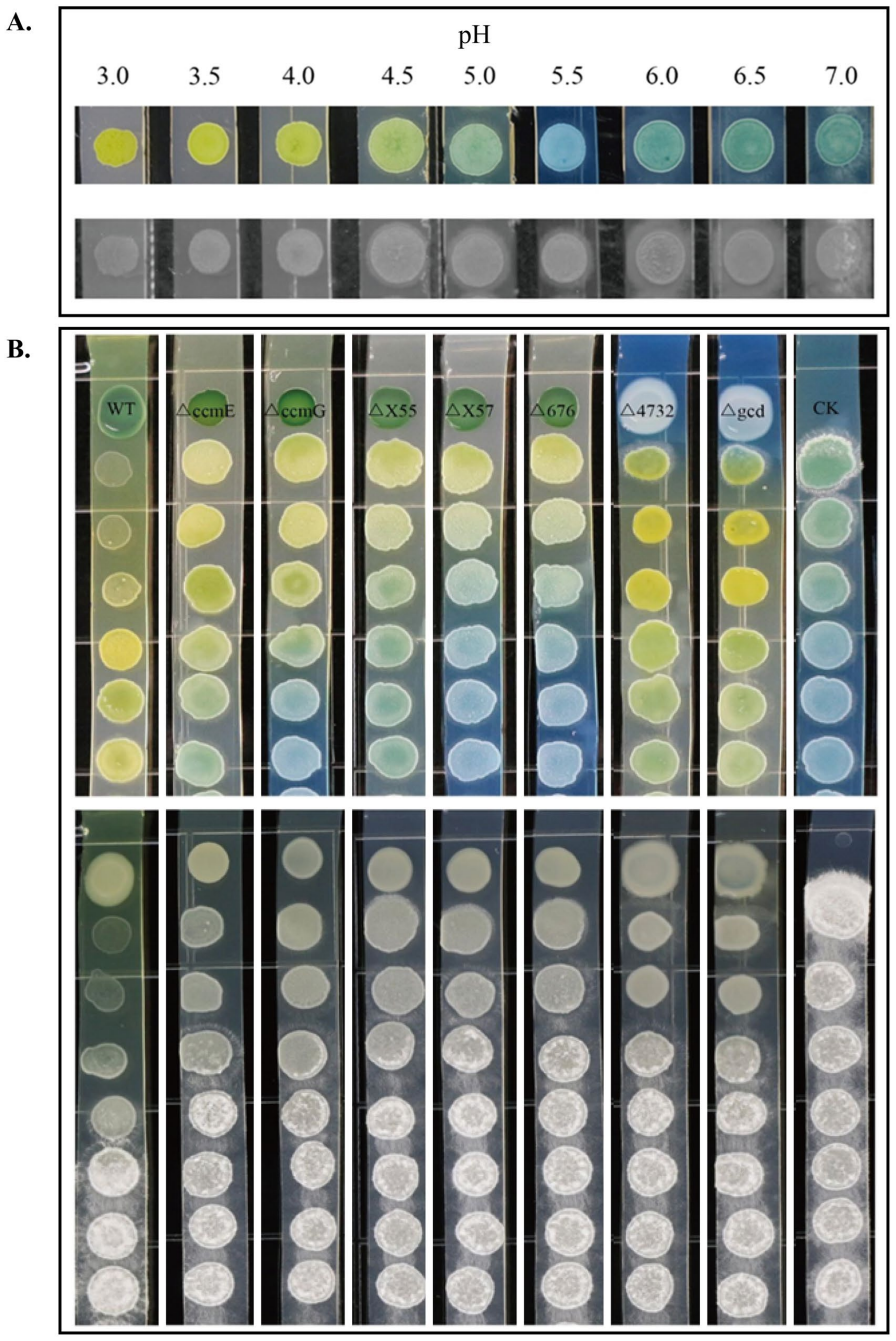
Role of environmental pH and the acidification of ST4 wild-type and Tn5 mutants in the inhibition of *S. scitamineum* sexual mating. **(A)** The direct effect of environmental pH on *S. scitamineum* sexual mating. MAT-1 and MAT-2 sporidia were co-cultured on PDA medium buffered to different pH levels. The top row shows the medium with bromocresol purple, which turns from yellow (acidic, pH ~ 3.5) to light green (neutral, pH ~ 7.0). The bottom row shows fungal growth on the same media without the indicator. Mycelial formation (white, fluffy growth) is severely inhibited under acidic conditions (pH ≤ 4.0) but occurs robustly at neutral pH. **(B)** Confrontation assays linking the acidification of wild-type ST4 and selected Tn5 mutants to antifungal activity. The strains were inoculated at one end of a PDA strip, with co-cultured *S. scitamineum* MAT-1 and MAT-2 spotted at intervals. The top row shows the medium acidification pattern visualized by bromocresol purple (yellow indicates acidity). The bottom row shows the corresponding inhibition of fungal mating.

Given the role of Gcd in catalyzing the oxidation of glucose to gluconic acid, we hypothesized that ST4 inhibits *S. scitamineum* mating by acidifying the extracellular environment. To test this, we first examined the direct effect of environmental pH on fungal sexual mating. MAT-1 and MAT-2 sporidia were co-cultured on PDA medium buffered to different pH levels, with bromocresol purple added as a visual pH indicator. Mycelial formation was severely inhibited under acidic conditions: at pH ≤ 3.5 (yellow medium), the fungal colonies remained yeast-like with no visible mycelial development. A slight increase to pH 4.0 (light yellow medium) allowed sparse mycelia to form only at colony peripheries. Mycelial production progressively increased with pH: moderate growth was observed between pH 4.5 ~ 5.5 (light blue medium), and robust, abundant myceliation occurred at neutral pH (6.0 ~ 7.0, light green medium) (Figure 4A). Notably, haploid sporidia remained viable and capable of growth under all tested pH conditions. These results demonstrate a clear correlation between environmental acidity and the inhibition of sexual mating in *S. scitamineum*, confirming that acidity alone is sufficient to block the dikaryotic transition essential for pathogenesis.

We next employed a confrontation assay to examine the correlation between environmental acidification and inhibitory activity in wild-type ST4 and selected Tn5-insertion mutants. Each bacterial strain was inoculated at one end of a PDA strip containing bromocresol purple, while MAT-1 and MAT-2 sporidia were co-inoculated at sequential points along the strip. Tested Tn5-insertion mutants included T*gcd* (the most frequently isolated mutant) and six other representative mutants (T*4732,* T*X57*, T*X55*, T*676*, T*ccmE*, T*ccmG*) that exhibited reduced inhibitory activity during initial screening. Results revealed a clear gradient of inhibitory activity that corresponded directly with the degree of medium acidification (Figure 4B). Wild-type ST4 completely inhibited sexual mating at the first three fungal inoculation spots and partially at the fourth. Mutants T*ccmE* and T*ccmG* showed moderate activity, inhibiting mating up to the second fungal inoculation spot. In contrast, mutants T*X57,* T*X55*, and T*676* only partially suppressed mating at the first spot, while T*gcd* and T*4732* completely lost inhibitory capacity.

This phenotypic gradient mirrored the observed acidification pattern in the medium (Figure 4B). A distinct yellow halo, indicating strong acidification, surrounded the wild-type ST4 inoculation site and extended across the first three fungal spots where mating was suppressed. The acidification and inhibition effects diminished with increasing distance from the bacterial colony. Mutants T*ccmE* and T*ccmG* produced a light-yellow halo, while T*X57,* T*X55*, and T*676* showed negligible color change, suggesting much reduced acidification. The non-inhibitory mutants T*gcd* and T*4732* were surrounded by a light blue halo, indicating a near-neutral environment (Figure 4B). These results demonstrate a strong positive correlation between a strain’s capacity to acidify its environment and its ability to inhibit fungal sexual mating.

To further confirm the causal relationship between these genetic loci and the biocontrol phenotype, we performed genetic and chemical complementation assays. The complementation of *gcd* and *pqq* in their respective knock-out mutant backgrounds fully restored their ability to inhibit *S. scitamineum* sexual mating (Supplementary Figure S2A). Furthermore, we investigated whether the loss of function in the *pqq* mutants could be rescued extracellularly. Exogenous supplementation of purified PQQ into the culture medium successfully restored the mating-inhibition capacities of the *pqq* mutants (Supplementary Figure S2B). Overall, our data strongly support the conclusion that PQQ-Gcd-mediated acidification constitutes an important component of the biocontrol mechanism employed by ST4.

### Distribution of ST4 biocontrol genes in Pseudomonadaceae

**Figure 5:**
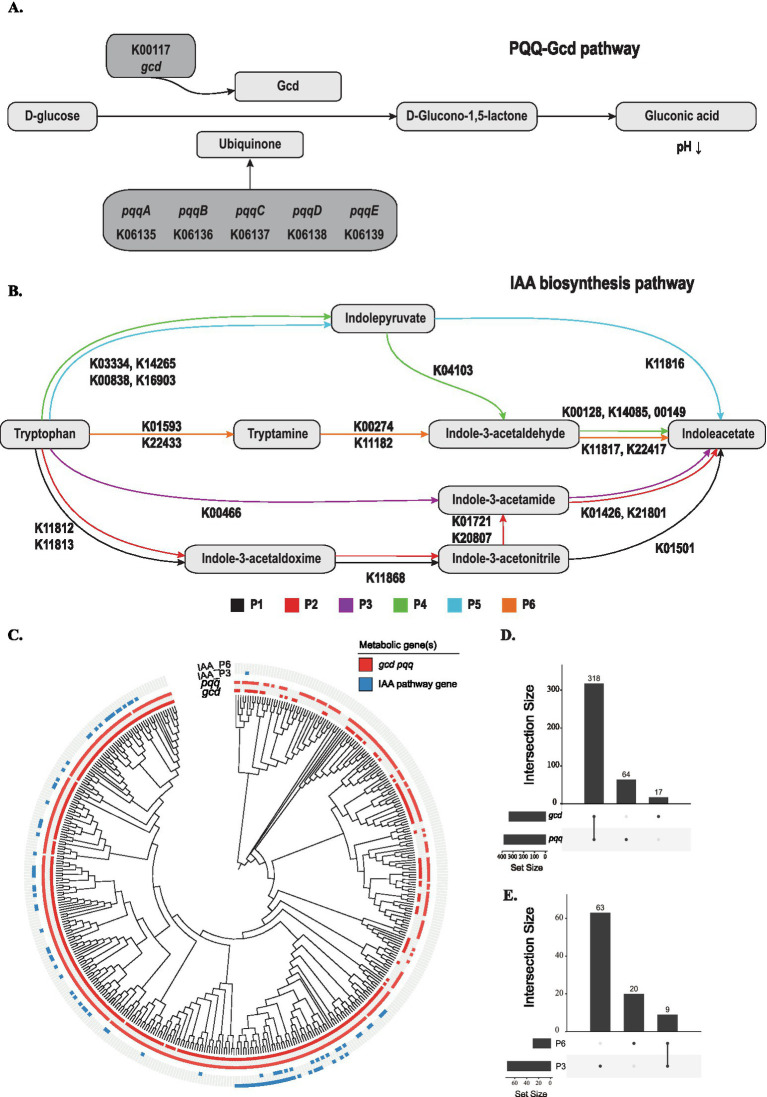
Genomic distribution of the PQQ-Gcd and IAA biosynthetic pathways across the Pseudomonadaceae family. **(A)** Schematic diagram of the PQQ-Gcd pathway for gluconic acid production, showing the biosynthesis of the pyrroloquinoline quinone (PQQ) cofactor by the *pqq* operon and its role in the glucose dehydrogenase (Gcd)-catalyzed oxidation of glucose to gluconic acid. **(B)** Schematic diagram of the bacterial biosynthesis pathways for indole-3-acetic acid (IAA), highlighting the key enzymes and intermediates. **(C)** Phylogenetic tree of Pseudomonadaceae with heatmaps indicating the presence (filled) or absence (open) of complete PQQ-Gcd and IAA biosynthesis pathways in 432 representative species. **(D)** Upset plots showing the combinations of *pqq* and *gcd* genes constituting a complete PQQ-Gcd system. **(E)** Upset plots showing the combinations for the indole-3-acetamide (IAM) and tryptamine (TAM) IAA biosynthesis pathways.

The production of gluconic acid via the PQQ-Gcd pathway (Figure 5A), a key mechanism identified in ST4, has been previously documented in various *Pseudomonas* species, suggesting it may represent a conserved functional strategy within this lineage. To systematically assess its distribution, we analyzed 432 representative genomes—one per species within the family Pseudomonadaceae—retrieved from the NCBI database. Functional annotation of their proteomes was performed using KofamKOALA from the KEGG database. Our analysis revealed that the complete genetic machinery for this pathway (*gcd* and the essential *pqqBCDE* genes) is present in 73.61% of all Pseudomonadaceae species, indicating a widespread capacity for PQQ-Gcd-mediated acidification (Figures 5C,D). An additional 17 species across the genera *Entomomonas*, *Halopseudomonas*, *Pseudomonas*, and *Stutzerimonas* possess the *gcd* gene but lack the *pqq* operon (Figures 5C,D). These strains may still retain Gcd activity by acquiring the PQQ cofactor exogenously from the environment, a form of metabolic “public good” cooperation.

Beyond acidification, our earlier work identified indole-3-carbaldehyde (ICHO) as an active metabolite produced by ST4. While the bacterial biosynthesis of ICHO remains poorly characterized, it is hypothesized to originate from the degradation of indole-3-acetic acid (IAA) or from intermediates of its biosynthesis. We therefore investigated the distribution of known bacterial IAA biosynthetic routes—the indole-3-acetamide (IAM), indole-3-pyruvate (IPyA), tryptamine (TAM), and indole (INA) pathways—across the Pseudomonadaceae (Figure 5B). KEGG-based annotation identified complete IAM and/or TAM pathways in 21.30% of the species, while the other IAA pathways were missing (Figures 5C,E). Notably, the genetic potential for IAA biosynthesis appears to be exclusively conserved within the genus *Pseudomonas* (Figure 5B). The co-occurrence of both the PQQ-Gcd acidification system and IAA-derived specialized metabolites like ICHO in *Pseudomonas* underscores the evolution of multifaceted and synergistic strategies for environmental adaptation and biocontrol within this genus.

## Discussion

The genus *Pseudomonas* encompasses a vast array of metabolically versatile bacteria that are dominant in diverse environments, particularly the rhizosphere (Palleroni, 2015). Certain species, such as *P. fluorescens* and *P. putida*, are renowned for their plant-beneficial properties, including growth promotion and biocontrol against phytopathogens (Mercado-Blanco and Bakker, 2007; Jatan et al., 2023). Our study introduces *Pseudomonas shiyinensis* sp. nov. ST4 as a novel member of this group, identified through robust genomic and chemotaxonomic analyses that clearly distinguish it from its closest relative, *P. guariconensis* (Toro et al., 2013). The complete genome sequence of ST4 reveals an extensive genetic repertoire for environmental adaptation and host interaction, including numerous genes associated with stress response, heavy metal detoxification, and secondary metabolite synthesis. Notably, the genomic blueprint confirms the presence of a complete pathway for glucose dehydrogenase (Gcd) synthesis, providing a molecular explanation for its previously documented glucose-dependent biocontrol activity (Liu et al., 2017). This work not only expands the taxonomic boundaries of the *Pseudomonas* genus but also provides a foundational genetic resource for elucidating the mechanistic underpinnings of ST4’s antifungal properties.

Our functional genomic and mutational analyses convergently identify PQQ-Gcd-mediated acidification as a central mechanism underlying ST4’s ability to suppress sexual mating in sugarcane smut. This discovery provides a direct genetic rationale for the previously reported glucose-dependent antifungal activity (Liu et al., 2017). The fact that 78% of the mapped loss-of-function mutations in our random Tn5 mutagenesis screen occurred within the *pqq* operon or the *gcd* gene itself, offers compelling evidence that this pathway serves as the primary mediator of mating inhibition. Subsequent *in vitro* pH assays confirmed that acidity alone is sufficient to recapitulate the biocontrol phenotype, severely impairing the dikaryotic transition essential for *S. scitamineum* pathogenesis while sparing the haploid sporidia. This specific inhibition of mating, rather than general fungicidal activity, presents a sophisticated strategy to suppress disease without imposing strong selective pressure for resistance (Jin et al., 2025). Confrontation assays with wild-type and mutant strains further demonstrated a strong correlation between a strain’s capacity to acidify its local environment and its ability to inhibit fungal mating.

While direct disruptions of the *pqq* and *gcd* genes caused most loss-of-function phenotypes, our Tn5 screen identified other loci whose disruption reduced inhibitory activity (Supplementary Figure S3), revealing that PQQ-Gcd-mediated acidification requires robust hierarchical regulation and systems-level integration. Upstream of the metabolic machinery, we identified an insertion in *gacS*, encoding the sensor kinase of the GacS/GacA two-component system. In *Pseudomonas*, the Gac system serves as a master global regulator of secondary metabolism and biocontrol traits; its disruption could affect the expression of the *pqq* operon. Furthermore, transposon insertions often exert polar effects, disrupting the transcription of adjacent genes. This is evident regarding the electron transport chain, as Gcd must transfer electrons to downstream carriers to function (Chukwubuikem et al., 2021). Insertions in the cytochrome *c* maturation operon (*ccmE* and *ccmG*) impair cytochrome *c* assembly, a primary electron acceptor (Kranz et al., 2009), halting gluconic acid production even with intact *gcd* and *pqq* genes. Similarly, an insertion in *pepP* sits upstream of *ubiH*, a gene essential for ubiquinone biosynthesis (Amir et al., 2017). Polar disruption here likely creates a bioenergetic bottleneck by depriving the chain of this vital electron carrier. Furthermore, substrate competition and carbon flux are critical. An insertion in *leuA* likely exerts a polar effect on downstream genes like *zwf*, *pgl*, and *hexR* (a master regulator of glucose metabolism) (Shah et al., 2022; Antunes et al., 2016). Altering these genes could redirect glucose into intracellular phosphorylation pathways, depleting the periplasmic substrate pool required by Gcd.

While our *in vitro* assays on PDA plates demonstrated a clear correlation between acidification and mating inhibition, it is important to consider the complexity of the soil environment. Bulk soil possesses a natural buffering capacity that could theoretically neutralize organic acids. However, the biocontrol interaction does not occur in bulk soil, but rather in the rhizosphere and colonization sites on sugarcane buds—niches rich in root exudates (glucose) that fuel the PQQ-Gcd pathway. We propose that strain ST4 creates a localized acidic micro-environment in the immediate vicinity of the infection court. This localized pH reduction is sufficient to disrupt the mating of *S. scitamineum* sporidia on the root or bud surface, despite the buffering capacity of the surrounding bulk soil.

The significance of this mechanism is underscored by its widespread conservation. Our phylogenomic analysis across the Pseudomonadaceae family reveals that the genetic capacity for PQQ-Gcd-mediated acidification is remarkably pervasive, being fully retained in over 73% of species. This prevalence suggests strong evolutionary selection for this trait, likely due to its dual utility in nutrient acquisition and ecological influences (e.g., microbial competition, host interaction, and resources utilization) (Cheng et al., 2015; Rodriguez and Fraga, 1999). The presence of the *gcd* gene alone in certain taxa further implies a potential for “public good” cooperation in microbial communities, wherein PQQ produced by one organism can be utilized by others (Jin et al., 2025). Such ecological flexibility could enhance the resilience and functional stability of this biocontrol strategy in complex soil environments. The PQQ-Gcd system thus emerges not merely as a strain-specific attribute of ST4, but as a major, evolutionarily conserved biocontrol module within this important bacterial family.

Beyond acidification, the genome of ST4 hints at a multi-layered biocontrol strategy. The production of indole-3-carbaldehyde (ICHO), a specific inhibitor of sugarcane smut mating, illustrates a complementary tactical approach (Liu et al., 2018). While the bacterial biosynthetic pathway for I3C remains incompletely mapped, its proposed derivation from the common phytohormone precursor tryptophan links it to the indole-3-acetic acid (IAA) biosynthesis pathway (Spaepen and Vanderleyden, 2011; Cui et al., 2022). Our analysis reveals that the capacity for IAA synthesis is a less common trait, confined to a subset of *Pseudomonas* species, highlighting a specialized adaptive strategy within the genus (Patten and Glick, 1996). We posit that ST4 employs a dual metabolic strategy: utilizing the widely conserved PQQ-Gcd system for broad-spectrum environmental acidification and deploying IAA-derived metabolites like ICHO for targeted disruption of fungal sexual reproduction. This combination of a general stressor (acidification) with a specific inhibitor (ICHO) could provide a powerful, synergistic advantage in suppressing rhizosphere diseases (Berg, 2009).

While both the PQQ-Gcd pathway and indole-3-carbaldehyde (ICHO) production are antifungal traits of ST4, our Tn5 transposon mutagenesis screen—which covered approximately 40,000 mutants—predominantly returned hits mapping to the *pqq* and *gcd* gene clusters, with no direct hits in ICHO biosynthetic genes affecting the mating inhibition phenotype under the tested conditions. This strongly suggests a hierarchical contribution of mechanisms: PQQ-Gcd-mediated acidification acts as the primary and dominant mechanism for mating inhibition, likely due to the rapid kinetics of gluconic acid production in high-glucose environments. ICHO likely functions as a supplementary or secondary mechanism, potentially relevant under conditions where glucose is limiting or as a broad-spectrum antibiotic, but it appears less critical for the specific phenotype of mating inhibition observed in this study.

The phylogenomic analysis of 432 Pseudomonadaceae genomes revealed a fascinating evolutionary pattern: while the *gcd* gene is highly conserved, the *pqq* biosynthetic operon is frequently lost in many lineages. This distribution points to a “public goods” evolutionary dynamic. PQQ is a secreted cofactor that can be shared within the microbial community. Strain ST4, which possesses the complete biosynthetic machinery, may function as a “keystone” or “helper” strain within the microbiome. By secreting PQQ, ST4 potentially activates the latent Gcd enzymes of neighboring PQQ-negative beneficial bacteria, thereby “unlocking” their ability to produce gluconic acid and solubilize phosphates. This implies that the application of ST4 could have synergistic effects, enhancing not only pathogen suppression but also the metabolic activity of the indigenous microbiome.

In conclusion, our integrated genomic and functional study elevates ST4 from a promising biocontrol isolate into the novel species *Pseudomonas shiyinensis* sp. nov. and established the molecular basis for its potent biocontrol activity. We have pinpointed PQQ-Gcd-mediated acidification as its principal and powerful mode of action, a trait that is both widespread and evolutionarily conserved among related bacteria. The concurrent presence of pathways for specialized metabolites like ICHO suggests a sophisticated, multi-mechanistic approach to biocontrol. These insights advance our understanding of microbial warfare in the rhizosphere and offer a clear roadmap for future applications. The genetic markers identified here can guide the screening of novel biocontrol strains, and the elucidated mechanisms can be leveraged for metabolic engineering to enhance the efficacy and reliability of next-generation biocontrol agents tailored for sustainable agriculture (Compant et al., 2005).

### Description of *Pseudomonas shiyinensis* sp. nov.

*Pseudomonas shiyinensis* (shi.yin.en’sis. N. L. masc./fem. Adj. shiyinensis, honoring the late Dr. Shiyin Liu, who first isolated and characterized the type strain ST4).

Cells are Gram-stain-negative, aerobic, and rod-shaped (0.6–0.8 μm wide and 1.5–2.5 μm long). Motile by means of polar flagella. Colonies on NA are circular, convex, smooth, and beige. Growth occurs at 10–44 °C (optimum 28 °C), pH 5.0–10.0 (optimum pH 7.0), and with 0–5% (w/v) NaCl. Positive for arginine dihydrolase, catalase, oxidase, and urease. Negative for nitrate reduction, indole production, and gelatin hydrolysis. In Biolog GN III assays, the strain utilizes D-glucose, D-fructose, glycerol, L-arginine, L-histidine, L-pyroglutamic acid, L-serine, L-aspartic acid, L-glutamic acid, L-lactic acid, citric acid, D-malic acid, α-keto-glutaric acid, L-malic acid, bromo-succinic acid, β-hydroxybutyrate, D-gluconic acid, L-butyric acid, propionic acid, and γ-amino-butyric acid. The major cellular fatty acids are C_16:0_, C_17:0_ cyclo, and C_19:0_ cyclo ω8c. The major respiratory quinone is Q-9. The polar lipid profile consists of diphosphatidylglycerol, phosphatidylethanolamine, phosphatidylglycerol.

The type strain, ST4^T^ (= CCTCC M2015526^T^), was isolated from vegetable rhizosphere in Shantou, Guangdong Province, China. The genome has a size of 5.14 Mb and a GC-content of 63.57 mol%. The GenBank accession number for the 16S rRNA gene sequence of strain ST4^T^ is KC634244. The complete genome sequence of ST4^T^ is associated with the BioProject PRJNA1385281.

## Methods and materials

### Bacterial strains, plasmids, and culture conditions

The strains and plasmids used in this study are listed in Supplementary Table S6. Strain ST4 and derivatives were routinely maintained at 28 °C in Luria-Bertani (LB) medium or minimal medium (MM) [10.5 g K_2_HPO_4_, 4.5 g KH_2_PO, 2.0 g (NH_4_)SO_4_, 0.2 g MgSO_4_·7H_2_O, 0.01 g CaCl_2_, 0.005 g FeSO_4_·7H_2_O, and 0.002 g MnCl_2_·4H_2_O, per L] with various carbon sources of the indicated concentrations. *E. coli* strains were routinely maintained in LB medium at 37 °C. The *S. scitamineum* haploid sporidia MAT-1 and MAT-2 were routinely maintained at 28 °C in YePS medium (20.0 g peptone, 10.0 g yeast extract, and 10.0 g sucrose, per L). PDA (200.0 g potato, 20.0 g glucose, and 18.0 g agar, per L) was used for bioassay of bacterial antagonistic activity against fungal mating. *Pseudomonas* isolation agar (PIA; 20.0 g peptone, 1.4 g MgCl_2_, 10.0 g K_2_SO_4_, 25 mg Irgasan, and 15.0 g agar, per L) was used for gene knock-out. The following antibiotics were supplemented when required: 50 μg/mL ampicillin, 50 μg/mL kanamycin, and 30 μg/mL gentamicin.

### Genome sequencing, assembly, and annotation

For whole genome sequencing, ST4 was cultured in Luria-Bertani (LB) medium at 28 °C for 12 h, and DNA was isolated using the MasterPure™ DNA Purification Kit (Epicentre Co., United States) according to the manufacturer’s instructions for gram-negative bacteria. DNA quality was monitored on 1% agarose gels, and DNA concentration was measured using a Nanodrop2000C spectrophotometer. The sequencing libraries were generated from a total amount of 0.2 μg genomic DNA using NEB Next® Ultra™ DNA Library Prep Kit for Illumina (NEB, United States) following the manufacturer’s recommendations. High-throughput sequencing was conducted by BGI (Wuhan, China) using the Illumina HiSeq 2500 and PacBio RS II platforms (Rhoads and Au, 2015).

Initial assembly was conducted using Canu v1.9 with the “pacbio-raw” option (Koren et al., 2017). The assembly was then polished with Illumina short reads using Pilon v1.23 through three iterative runs with the “-fix snps, indels” parameter (Walker et al., 2014). Assembly completeness was assessed using both Benchmarking Universal Single-Copy Orthologs (BUSCO) v5.5.0 (with the Pseudomonadales_odb10 gene set) (Manni et al., 2021) and CheckM v1.2 (Parks et al., 2015). To assess assembly quality, Illumina and PacBio reads were mapped to the assembly using BWA v0.7.17 (Li and Durbin, 2009) and Minimap2 v2.17 (Li, 2018), respectively, with statistics generated using Samtools v1.9 flagstat (Li et al., 2009). Finally, the circular genome plot was generated using GenoVi v0.2.16 (Cumsille et al., 2023).

The genome annotation was generated using the NCBI Prokaryotic Genome Annotation Pipeline (PGAP) version 2024-04-27.build7426 (Tatusova et al., 2016). Potential secondary metabolite biosynthesis gene clusters, plant growth promoting genes, preferred habitats, and virulence factors of ST4 were predicted using antiSMASH v7.1.0 (Blin et al., 2023), PGPg_finder v1.1.0 (Pellegrinetti et al., 2024), ProkAtlas server^1^ (Mise and Iwasaki, 2020), and VFanalyzer^2^ (Liu et al., 2019), respectively.

### Phylogenomic analysis and comparative genomics

Representative and type strain genome sequences of all *Pseudomonas* species were retrieved from the NCBI Genome database. The Average Nucleotide Identity (ANI) values between ST4 and each of the *Pseudomonas* genomes were initially calculated using skani v0.2.2 (Shaw and Yu, 2023). For the most similar *Pseudomonas* genomes identified in this initial screen, their ANI values with ST4 were further verified using pyani v0.2.13 with the “ANIb” method (Pritchard et al., 2016), and digital DNA–DNA hybridization (dDDH) values were calculated using the Genome-to-Genome Distance Calculator (GGDC) server^3^ (Meier-Kolthoff et al., 2013). A whole-genome-based phylogenetic tree was reconstructed using tANI_tool v1.3.0 with default settings (Gosselin et al., 2022), and the reliability of the tree topology was estimated by 100 bootstrap replicates. The resulting phylogenetic tree alongside ANI and dDDH values were visualized using Interactive Tree Of Life (iTOL) (Letunic and Bork, 2021). Pairwise whole-genome alignments between ST4 and *P. guariconensis*, *P. plecoglossicida, P. inefficax, P. fakonensis,* and *P. mosselii* were generated using MUMmer v4.0.1 (Marçais et al., 2018).

### Chemotaxonomy

For chemotaxonomic characterization, ST4 was cultured on LB plates at 28 °C for 48 h. Cellular fatty acid methyl esters were extracted from 100 mg of fresh cells and analyzed using the Sherlock Microbial Identification System (Marçais et al., 2018). Polar lipids were extracted and analyzed by two-dimensional thin-layer chromatography (Minnikin et al., 1984). Respiratory quinones were extracted and analyzed by high-performance liquid chromatography (Collins, 1985). All chemotaxonomic analyses were conducted at the Guangdong Institute of Microbiology (Guangdong, China).

### Tn5 mutant screening

Mutants with lost inhibitory effects on the sexual mating of *S. scitamineum* haploid strains MAT-1 and MAT-2 were obtained using a biparental conjugation method. *Escherichia coli* S17-1 strains (Simon et al., 1983) carrying pTGN plasmid with Tn5 transposons and Gentamicin resistance gene were cultured overnight on LB plates at 37 °C, while recipient wild-type ST4 strains were cultured overnight on LB plates at 28 °C. Donor and recipient strains were washed from plates using minimal medium (MM) and concentrations were adjusted to the same OD_600_. Equal volumes of both strains were combined and spotted onto LB plates, then incubated at 28 °C for 6 h. Transconjugants were washed using MM medium and spread onto MM plates containing 50 μg/mL gentamicin and ampicillin, followed by incubation at 28 °C. After colony growth, bacteria exhibiting different morphologies were selected for inhibition effect detection. Mutants that lost the ability to inhibit sexual mating were preserved for further analysis.

Frozen MAT-1 and MAT-2 strains were streaked onto YEPS plates containing gentamicin (50 μg/mL) and ampicillin (100 μg/mL), then incubated at 28 °C for 2–3 days. Single colonies were cultured in liquid YEPS medium with shaking at 200 rpm until OD₆₀₀ reached 1.5. MAT-1 and MAT-2 strains were then mixed in a 1:1 ratio. For screening Tn5 mutants, 30 mL of PDA medium (pH 6.1) was poured into a 12 × 12 cm square Petri dish. After solidification, the medium was cut into 0.6 × 6 cm strips. To investigate pH effects, PDA medium was adjusted to pH values from 3.0 to 7.0, with bromocresol purple (40 μg/mL) added as a pH indicator. A 0.5 μL aliquot of bacterial suspension was applied to the end of each PDA strip, and the prepared mixture of *S. scitamineum* haploid strains was spotted along the strips (0.5 μL per spot, with 0.5 cm intervals between spots). After air-drying, plates were inverted and incubated in the dark at 28 °C for 1–2 days.

### Obtaining flanking sequences of Tn5 insertion

Genomic DNA was extracted from wild-type ST4 and mutants using the MasterPure™ DNA Purification Kit. Initial screening used primers GT-F (5′-GCAGTCGCCCTAAAACAAA-3′) and GT-R (5′-CACTTCTTCCCGTATGCCCAACTT-3′) designed based on the gentamicin resistance gene from plasmid pTGN. Flanking sequences of Tn5 insertion sites were obtained using hiTAIL-PCR with transposon-specific primers (G1, G2, G3) and random degenerate primers (LAD1-4, AC1; Supplementary Table S7) (Liu and Chen, 2007). Third-round hiTAIL-PCR products were purified, cloned into T-vectors, and transformed into *E. coli*. Positive clones were identified by PCR and sequenced. The obtained flanking sequences were aligned to the ST4 genome using BLAST to identify disrupted genes (Altschul et al., 1990).

### Construction of in-frame deletion mutants and complementary strains

To knock out the target genes, the upstream and downstream DNA fragments were amplified using primer pairs -UF/-UR and -DF/-DR listed in Supplementary Table S7 with Phanta Super-Fidelity DNA Polymerase (Vazyme Biotech, Nanjing, China). The fragments were then fused and ligated into *Bam*HI/*Eco*R1-digested suicide vector pK18mob*sacB* using the C112-01 ClonExpress II One Step Cloning Kit (Vazyme Biotech, Nanjing, China). The recombinant vector was transformed into competent *E. coli* DH5α cells and subsequently transferred to wild-type cells via triparental mating with the help of plasmid pRK2013. Colonies resistant to 10% sucrose were selected on PIA agar plates and verified by PCR using the -F/-R primers (Supplementary Table S7). The deletion of genes was confirmed through sequencing.

For gene complementation, the open reading frames (ORF) was amplified by PCR using primers listed in Supplementary Table S7. PCR fragments were ligated into the downstream of the *lac* promoter of pBBR1-MCS5 between the *Hind*III and *Spe*I sites. The plasmids were verified by PCR and DNA sequencing and introduced into ST4 strains by tri-parental mating with the helper strain *E. coli* HB101 (pRK2013). The transformants were selected on LB agar containing 30 μg/mL gentamicin, and the correct complementary strains were verified by PCR and DNA sequencing.

### Distribution of glucose dehydrogenase and auxin biosynthesis pathways in Pseudomonadaceae

Annotated proteomes of 432 Pseudomonadaceae representative genomes were obtained from the NCBI Genome database. Functional annotation of the protein sequences was performed using KofamScan v1.3.0 (Aramaki et al., 2020) with the KOfam database version 2025-02-01. The KO identifiers associated with the PQQ biosynthesis pathway, glucose dehydrogenase, and various IAA biosynthesis pathways were retrieved from the KEGG database (Kanehisa et al., 2023). A genome is considered to possess a complete pathway if at least one corresponding KO identifier was annotated for every step in the pathway.

## Data Availability

The datasets presented in this study can be found in online repositories. The names of the repository/repositories and accession number(s) can be found in the article/Supplementary material.

## Footnotes

1 Accessed at http://prokatlas.bs.s.u-tokyo.ac.jp/.

2 Accessed at http://www.mgc.ac.cn/cgi-bin/VFs/v5/main.cgi?func=VFanalyzer.

3 Accessed at https://ggdc.dsmz.de/.
