# Correction: Genomic and functional characterization of *Pseudomonas shiyinii* sp. nov. ST4 reveals conserved biocontrol mechanisms against sugarcane smut

**DOI:** 10.3389/fmicb.2026.1910926

**Published:** 2026-07-09

**Authors:** Yonglin Li, Shiyin Liu, Changlong Yang, Changqing Chang, Ming Hu, Lianhui Zhang, Jianuan Zhou, Xiaofan Zhou

**Affiliations:** National Key Laboratory of Green Pesticide, Guangdong Provincial Key Laboratory of Microbial Signals and Disease Control, Engineering Research Center of Biological Control, Ministry of Education, Integrative Microbiology Research Center, South China Agricultural University, Guangzhou, China

**Keywords:** biological control, glucose dehydrogenase, indole-3-acetic acid, novel species, *Pseudomonas*, sugarcane smut

The error was made in the Section **Results**, *Description of Pseudomonas shiyinensis sp. nov*., paragraph three, page 10.

The GenBank accession number for the 16S rRNA gene sequence of strain ST4^T^ was mistakenly displayed as “KC634244.”

The correct statement is below:

“The GenBank accession number for the 16S rRNA gene sequence of strain ST4^T^ is KU496562.”

