# Supplementary material for: Genomic and Functional Characterization of Pseudomonas shiyinii sp. nov. ST4 Reveals Conserved Biocontrol Mechanisms Against Sugarcane Smut
Source: Front Microbiol. 2026 May 20;17:1783009. doi: 10.3389/fmicb.2026.1783009 (PMC13231928; doi:10.3389/fmicb.2026.1783009)
Supplement: Supplementary file 1 [file Table_1.docx]

**
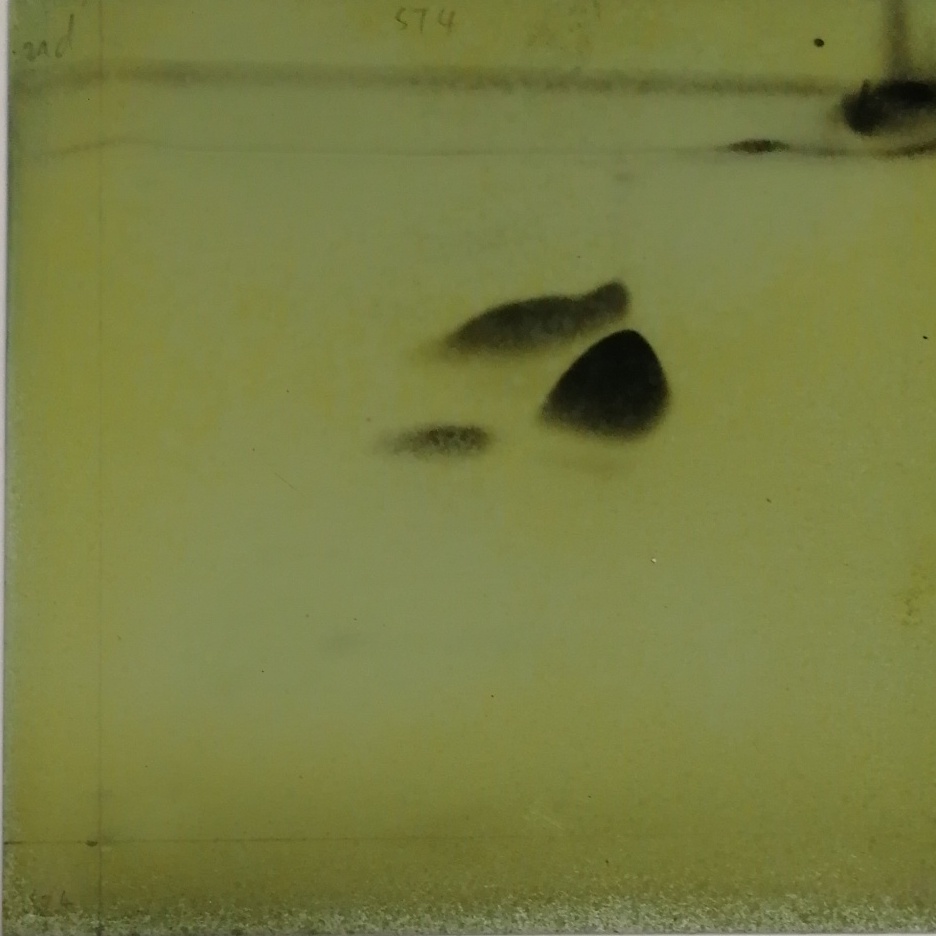
**

L

PG

PE

DPG

**A**

**B**


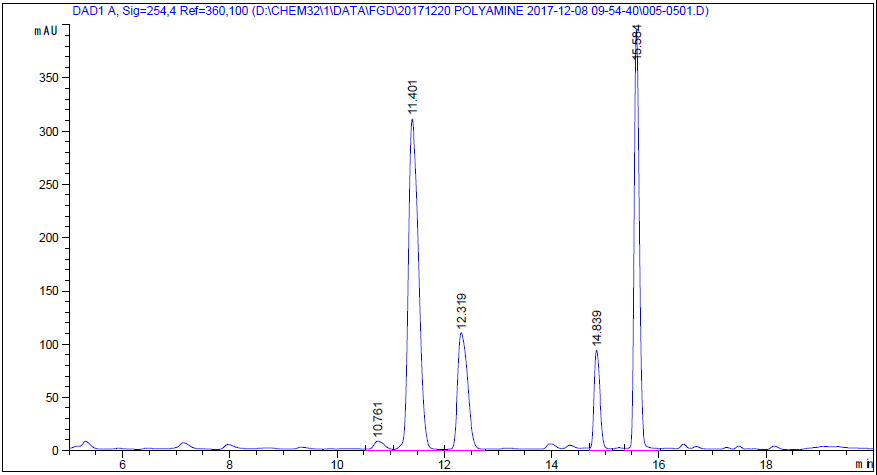


**1, 8-Dia**

**Spd**

**Put**

**Cad**

**Figure S1. Chemotaxonomy characterization of *Pseudomonas shiyinensis* sp. nov. ST4.** (A) Polyamine composition of ST4. 1,8-Dia was added as an internal standard. Abbreviations: Spd, Spermidine; Put, Putrescine; Cad, Cadaverine; 1,8-Dia, 1,8-Diaminooctane. (B) Two-dimensional chromatography of polar lipids of ST4. Abbreviations: DPG, diphosphatidylglycerol; PG, phosphatidylglycerol; PE, phosphatidylethanolamine; L, unidentified lipid.

**
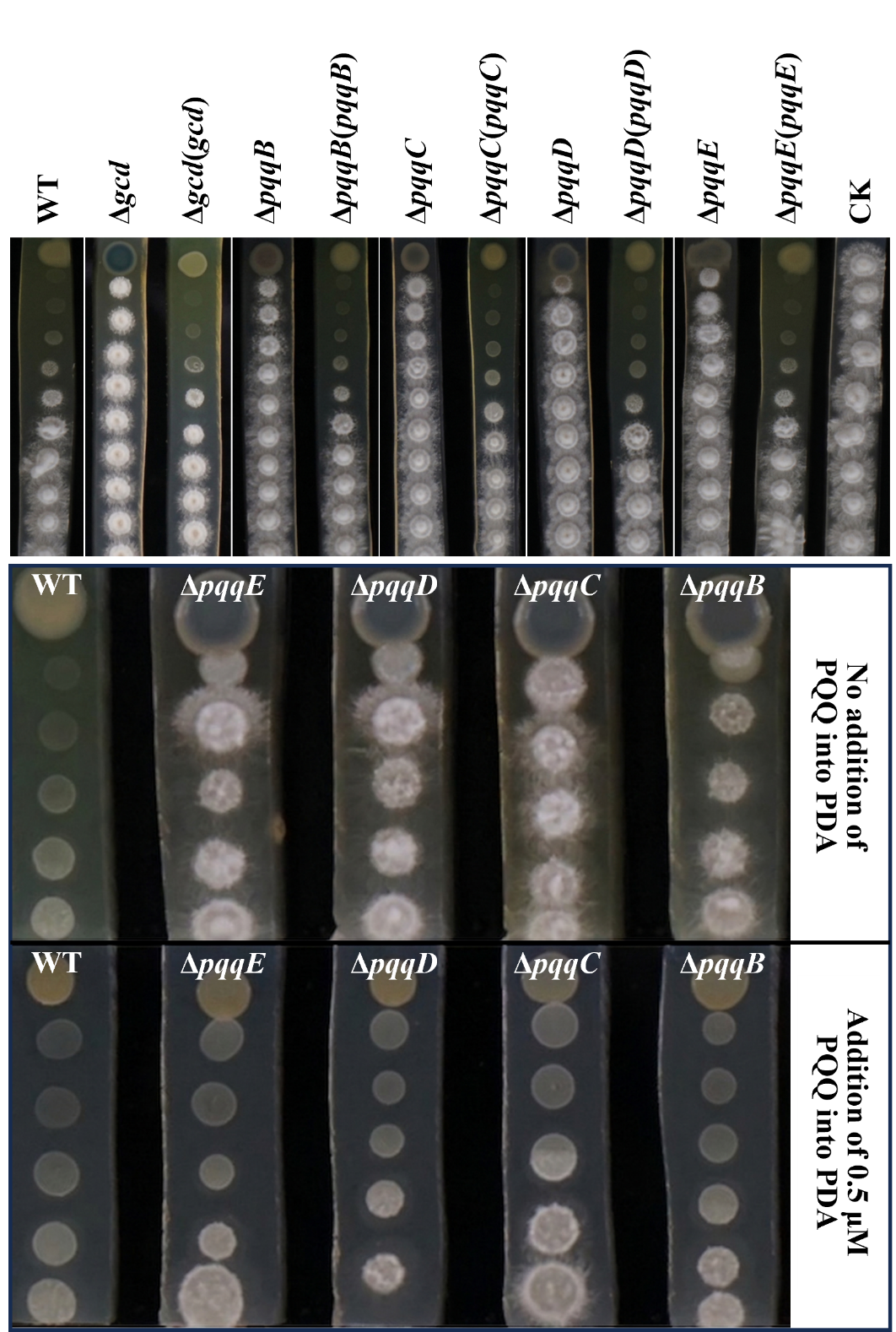
**

**B**

**A**

**Figure S2. Genetic and chemical complementation confirm the essential role of the PQQ-Gcd pathway in the biocontrol activity of ST4.** (A) Agar-plate confrontation assays displaying the mating inhibitory phenotype of wild-type (WT) ST4, control (CK), knockout mutants (Δ*gcd*, Δ*pqqB*, Δ*pqqC*, Δ*pqqD*, Δ*pqqE*), and their respective complementation strains (Δ*gcd*(*gcd*), Δ*pqqB*(*pqqB*), Δ*pqqC*(*pqqC*), Δ*pqqD*(*pqqD*), Δ*pqqE*(*pqqE*)). **(B)** Confrontation assays showing that exogenous supplementation of pyrroloquinoline quinone (PQQ) chemically rescues the mating inhibition defect of Δ*pqq* mutants.


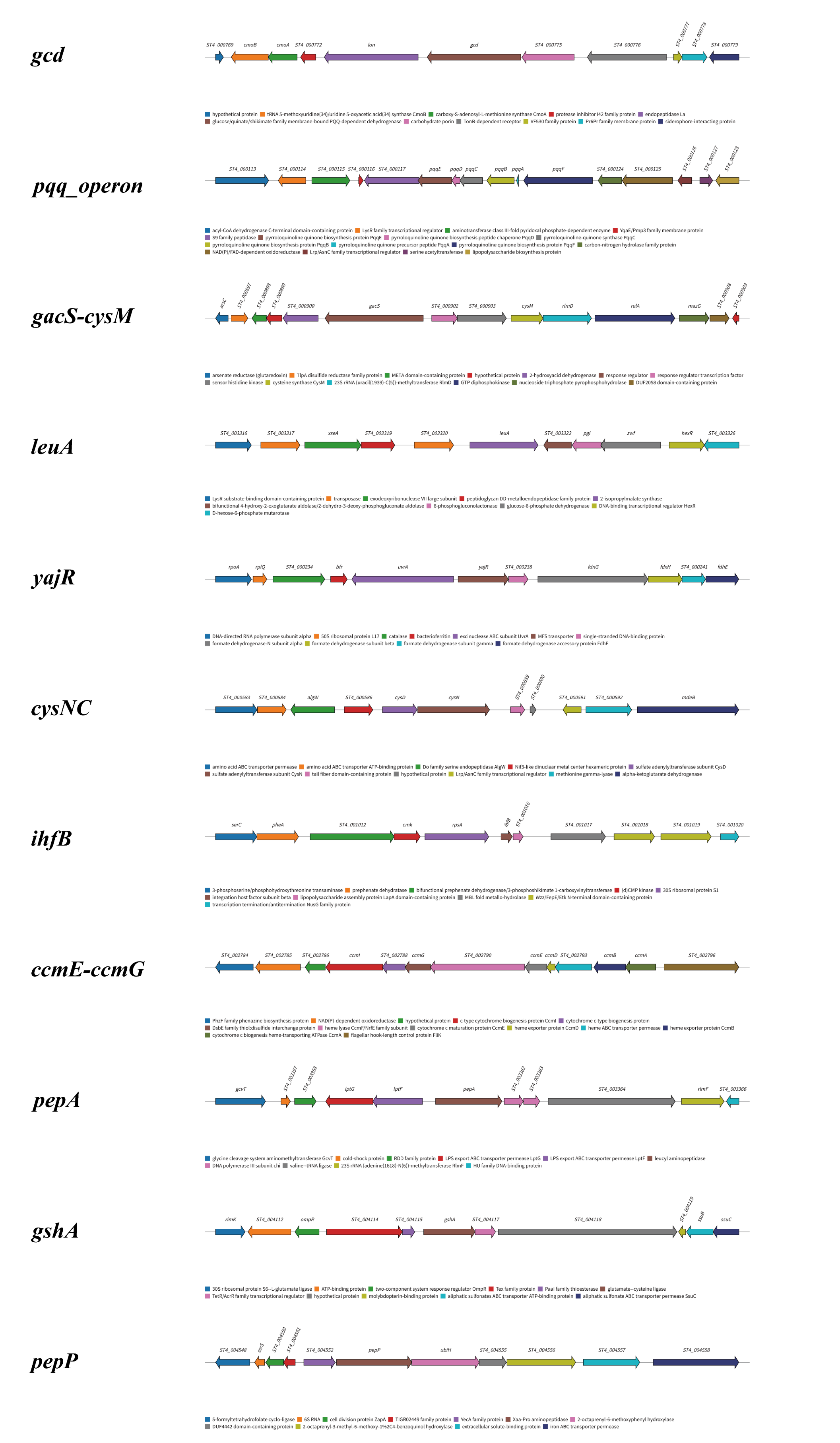


**Figure S3. Genomic context of the loci disrupted in the Tn5 mutagenesis screen.** The schema illustrates the local genomic neighborhoods of the genes whose disruption by Tn5 insertion resulted in abolished or reduced inhibitory activity of *Pseudomonas shiyinensis* ST4 against the sexual mating of *Sporisorium scitamineum*.

**Table S1. Secondary metabolite biosynthetic gene clusters identified in ST4.**

| **Region** | **Type** | **From** | **To** | **Most similar known cluster** | **Similarity** |
| --- | --- | --- | --- | --- | --- |
| Region 1 | redox-cofactor | 117,112 | 139,271 | lankacidin C | 13% |
| Region 2 | NAGGN | 1,038,851 | 1,053,746 | NA | NA |
| Region 3 | NRP-metallophore, NRPS | 1,454,281 | 1,520,685 | Pf-5 pyoverdine | 12% |
| Region 4 | NI-siderophore | 1,624,551 | 1,657,207 | vibrioferrin | 100% |
| Region 5 | RiPP-like | 1,888,351 | 1,899,184 | NA | NA |
| Region 6 | NRP-metallophore, NRPS | 1,916,045 | 1,993,371 | Pf-5 pyoverdine | 14% |
| Region 7 | RiPP-like | 2,750,303 | 2,762,507 | NA | NA |
| Region 8 | arylpolyene | 4,406,209 | 4,449,825 | APE Vf | 35% |
| Region 9 | RiPP-like | 4,817,186 | 4,827,965 | NA | NA |
| Region 10 | NRPS-like | 4,977,026 | 5,020,457 | nematophin | 13% |

**Table S2. Summary of plant growth-promoting genes identified in ST4.**

| **Functional category** | **Gene Count** |
| --- | --- |
| Osmotic Stress Neutralization | 245 |
| Motility Chemotaxis | 151 |
| Heavy Metal Detoxification | 145 |
| Surface Attachment | 116 |
| Vitamin Production | 102 |
| Fe-Siderophores | 65 |
| Organic Volatiles | 53 |
| Secretion Systems | 32 |
| Phytohormone-Cytokinins | 31 |
| Phytohormone-GABA | 29 |
| Phytohormone-IAA | 27 |
| Nitrogen Fixation | 24 |
| Root Colonization | 23 |
| Carbon Dioxid Fixation | 20 |
| P-Solubilization Gluconic Acid-PQQ | 14 |
| Urea Metabolism | 9 |
| P-Mineralization | 8 |
| Nitrate Reduction | 7 |
| Insecticidal Compounds | 6 |
| Denitrification | 4 |
| Nematicidal Compounds | 0 |

**Table S3. Potential habitats of ST4 predicted by ProkAtlas.**

| **Habitat** | **Preference score^*^** |
| --- | --- |
| Rhizosphere | 26.01694 |
| Soil | 9.428133 |
| Freshwater | 9.013025 |
| Biosolids | 5.855687 |
| Food | 5.643928 |
| Marine | 5.005989 |
| Wastewater | 3.363703 |
| Subsurface | 2.923874 |
| Fungus | 2.793702 |
| Sediment | 2.735546 |
| Terrestrial | 2.264939 |
| Groundwater | 1.974233 |
| Plant | 1.927999 |
| Viral | 1.738398 |
| Activated_sludge | 1.58509 |
| Bioreactor | 1.471911 |
| Marine_sediment | 1.276988 |
| Rock_porewater | 1.112503 |

^*^Only the habitats with preference score greater than 1 are shown.

**Table S4. Potential virulence factor encoding genes identified in ST4.**

| **Functional category** | **Gene Count** |
| --- | --- |
| Adherence | 66 |
| Iron uptake | 25 |
| Secretion system | 23 |
| Antiphagocytosis | 22 |
| Regulation | 7 |
| Immune evasion | 6 |
| Stress adaptation | 4 |
| Toxin | 2 |
| Efflux pump | 2 |
| Serum resistance | 2 |
| Quorum sensing | 1 |
| Acid resistance | 1 |
| Biofilm formation | 1 |
| Fimbrial adherence determinants | 1 |
| Invasion | 1 |
| Lipid and fatty acid metabolism | 1 |

**Table S5. Cellular fatty acid composition of ST4**^T^**.**

| **Fatty acids** | **Percent (%)** |
| --- | --- |
| 10:0 | 0.12 |
| 10:0 3OH | 4.87 |
| 12:0 | 2.10 |
| 11:0 3OH | 0.07 |
| 12:0 2OH | 7.23 |
| 12:0 3OH | 5.03 |
| 14:0 | 0.78 |
| 16:0 | 30.50 |
| 17:1 w7c | 0.19 |
| 17:0 cyclo | 23.48 |
| 17:0 | 0.25 |
| 18:0 | 0.58 |
| 18:1 w7c 11-methyl | 0.33 |
| 19:0 iso | 0.65 |
| 19:0 cyclo w8c | 12.28 |
| 20:2 w6,9c | 0.21 |
| Summed Feature 3^*^ | 3.65 |
| Summed Feature 8^**^ | 7.68 |

^*^ Summed Feature 3 comprises 16:1 w7c/16:1 w6c;

^**^Summed Feature 8 comprises 18:1 w7c.

**Table S6.** Bacterial strains and plasmids used in this study

| **Strain** | **Characteristic** | **Source or reference** |
| --- | --- | --- |
| ***Pseudomonas* strains** |  |  |
| P. shiyinensis ST4 | Wild-type strain, Amp^r^ | Liu et al., 2017 |
| T*ccmE* | ST4 with Tn5 insertion in *ccmE*, Amp^r^, Gen^r^ | This Study |
| T*ccmG* | ST4 with Tn5 insertion in *ccmG*, Amp^r^, Gen^r^ | This Study |
| T*X55* | ST4 with Tn5 insertion in *cysM*, Amp^r^, Gen^r^ | This Study |
| T*X57* | ST4 with Tn5 insertion in *gacS*, Amp^r^, Gen^r^ | This Study |
| T*X676* | ST4 with Tn5 insertion in *gshA*, Amp^r^, Gen^r^ | This Study |
| T*4732* | ST4 with Tn5 insertion in *pepA*, Amp^r^, Gen^r^ | This Study |
| T*gcd* | ST4 with Tn5 insertion in *gcd*, Amp^r^, Gen^r^ | This Study |
| Δ*gcd* | ST4 with *gcd* gene deleted | This Study |
| Δ*gcd*(*gcd*) | Δ*gcd* containing the expression constructs pBBR1-MCS5*-gcd* | This Study |
| Δ*pqqB* | ST4 with *pqqB* gene deleted | This Study |
| Δ*pqqB*(*pqqB*) | Δ*pqqB* containing the expression constructs pBBR1-MCS5*-pqqB* | This Study |
| Δ*pqqC* | ST4 with *pqqC*gene deleted | This Study |
| Δ*pqqC*(*pqqC*) | Δ*pqqC* containing the expression constructs pBBR1-MCS5*-pqqC* | This Study |
| Δ*pqqD* | ST4 with *pqqD* gene deleted | This Study |
| Δ*pqqD*(*pqqD*) | Δ*pqqD* containing the expression constructs pBBR1-MCS5*-pqqD* | This Study |
| Δ*pqqE* | ST4 with *pqqE* gene deleted | This Study |
| Δ*pqqE*(*pqqE*) | Δ*pqqE* containing the expression constructs pBBR1-MCS5*-pqqE* | This Study |
| ***Sporisorium scitamineum*** |  |  |
| *MAT-1* | haploid sporidia of *S. scitamineum* | Jia et al., 2025 |
| *MAT-2* | haploid sporidia of *S. scitamineum* | Jia et al., 2025 |
| ***E. coli* strains** |  |  |
| DH5α | *spuE44* Δ*lacU169*(*φ80lacZ*Δ*M15*) *hsdR17λpir* *recA1 endA1 gyrA96 thi-1 relA1* | Lab collection |
| pRK2013 | Tra^+^, Mob-, ColE1-replicon, Kan^r^, Spe^r^ | Lab collection |
| **Plasmids** |  | Lab collection |
| pK18mob*sacB* | *sacB*-based gene replacement vector, Gen^r^ | Lab collection |
| pBBR-MCS5 | Broad-host-range cloning vector; Gen | Lab collection |

Shiyin Liu, Nuoqiao Lin, Yumei Chen, Zhibin Liang, Lisheng Liao, Mingfa Lv, Yufan Chen, Yingxin Tang, Fei He, Shaohua Chen, Jianuan Zhou, and Lianhui Zhang, Biocontrol of Sugarcane Smut Disease by Interference of Fungal Sexual Mating and Hyphal Growth Using a Bacterial Isolate. Frontiers in Microbiology, 2017. 8:778.

Huan Jia, Rongrong Wu, Lei Li, Li Zhang, Xian Sun, Xiaonan Feng, Yifan Wang, Enping Cai, Shuquan Sun, and Changqing Chang, Induced cuproptosis by targeting the ESCRT-III complex potentiates copper-based control of smut diseases. International Journal of Biological Macromolecules, 2025. 305:141292.

**Table S7. Primers for hiTAIL-PCR**

| **Primer** | **Sequence (5' to 3')** | **Description** |
| --- | --- | --- |
| gcd-up-F | ctatgacatgattacgaattcCGCTGGCCTCGGACCAGT | Deletion of  *gcd* |
| gcd-up-R | aacgttactcggcCAGCACGCCCAGAAGGCG |  |
| gcd-dn-F | cgtgctgGCCGAGTAACGTTCCCGAG |  |
| gcd-dn-R | caggtcgactctagaggatccATGAAGAACAGCGCCAGGG |  |
| gcd-F | CAACGACGAACTCTACGCCC |  |
| gcd-R | GATGGTGCTTGAGCCAGGTG |  |
| pqqB-up-F | ctatgacatgattacgaattcAGGATTGTTACGGAGACTGCAATG | Deletion of  *pqqB* |
| pqqB-up-R | tcctAACTGCGCGCGGATGTCC |  |
| pqqB-dn-F | acatccgcgcgcagttAGGACCTGCGCACCGGCG |  |
| pqqB-dn-R | caggtcgactctagaggatccGGCGGACTCCTGTTATGGC |  |
| pqqB-F | CCTCATGCAGCGGTCTATCC |  |
| pqqB-R | CTGGCGGGTTGTGTAGTGCT |  |
| pqqC-up-F | ctatgacatgattacgaattcTCGAGGTGTTGGAGGGCTT | Deletion of  *pqqC* |
| pqqC-up-R | aactgcaggatAGTTGGCCAGGATCGCCG |  |
| pqqC-dn-F | tggccaactATCCTGCAGTTCAAGCTGGATATC |  |
| pqqC-dn-R | caggtcgactctagaggatccTCCAGCGGGTTGGAACAA |  |
| pqqC-F | AAGCTGTTCTACGCCCCAGG |  |
| pqqC-R | AATGCGGCGATCTTCTGCTC |  |
| pqqD-up-F | ctatgacatgattacgaattcGACGGCGCCCCGGGCGAG | Deletion of  *pqqD* |
| pqqD-up-R | cacgAGGGCGATCCCCTTGTGC |  |
| pqqD-dn-F | acaaggggatcgccctCGTGCCGGCCAAGCCCGA |  |
| pqqD-dn-R | caggtcgactctagaggatccCGATCTTGTCGATGTTGTGCC |  |
| pqqD-F | GGGTGGCCAATCGCTTCTAC |  |
| pqqD-R | TCTCGCGGTAGTCGTTGGTG |  |
| pqqE-up-F | ctatgacatgattacgaattcGCACCCGCCTGGGCCAGG | Deletion of  *pqqE* |
| pqqE-up-R | aatcggcttccagggcGTCAGGCGAGGACGATCCA |  |
| pqqE-dn-F | tgacGCCCTGGAAGCCGATTTC |  |
| pqqE-dn-R | caggtcgactctagaggatccTCGGCCGACTTGGCGCAC |  |
| pqqE-F | ACGCCTACGTCAACTTCGCC |  |
| pqqE-R | TCTTCGATGGTCTGCTGGGC |  |
| c-gcd-F | gcggtggcggccgctctagaactagtCAATGGTAGACGAAGGGGAG | Complement  of *gcd* |
| c-gcd-R | tcgaggtcgacggtatcgataagcttCCAGAGCAGTTTCTAACCCC |  |
| c-*pqqB*-F | gcggtggcggccgctctagaactagtATGTACATCCAGATCCTCGG | Complement  of *pqqB* |
| c-*pqqB*-R | tcgaggtcgacggtatcgataagcttTTACAACTCGATACTCAT |  |
| c-*pqqC*-F | gcggtggcggccgctctagaactagtATGAGCGACGCACTGCCGAT | Complement  of *pqqC* |
| c-*pqqC*-R | tcgaggtcgacggtatcgataagcttTCATAGGGCGATCCCCTTGT |  |
| c-*pqqD*-F | gcggtggcggccgctctagaactagtATGAGTTTCGACCGTCAACA | Complement  of *pqqD* |
| c-*pqqD*-R | tcgaggtcgacggtatcgataagcttTCAGGCGAGGACGATCCAGT |  |
| c-*pqqE*-F | gcggtggcggccgctctagaactagtGTGCCGGCCAAGCCCGAGGT | Complement  of *pqqE* |
| c-*pqqE*-R | tcgaggtcgacggtatcgataagcttTCAGCCTCGGGCAATGACAT |  |
| G1 | AACGCGCTTGGTGCTTATGT | hiTAIL-PCR |
| G2 | ACGATGGACTCCAGTCCGGCCATACAAAGTTGGGCATACG |  |
| G3 | CGACCCAAGTTCCGCCACC |  |
| LAD1 | ACGATGGACTCCAGAGCGGCCGC(G/C/A)N(G/C/A)NNNGGAA |  |
| LAD2 | ACGATCCACTCCAGAGCGGCCGC(G/C/T)N(G/C/T)NNNGGTT |  |
| LAD3 | ACGATGGACTCCAGAGCGGCCGC(G/C/A)(G/C/A)N(G/C/A)NNNCCAA |  |
| LAD4 | ACGATGGACTCCAGAGCGGCCGC(G/C/T)(G/A/T)N(G/C/T)NNNCGGT |  |
| AC1 | ACGATGGACTCCAGAG |  |
